# Graft to vein fistula with partial thrombosis: a case report

**DOI:** 10.3389/fcvm.2026.1654220

**Published:** 2026-03-26

**Authors:** Bo Hu, Zhen-Nan Liao, Guo-Liang Li, Zi-Ming Wan

**Affiliations:** 1 Department of Nephrology, The First Affiliated Hospital of Jinan University, Guangzhou, China; 2 People's Hospital of Tibet Autonomous Region, Lasa, China; 3 Department of Nephrology, The First Affiliated Hospital of Chongqing Medical University, Chongqing, China

**Keywords:** arteriovenous graft, graft to vein fistula, hemodialysis, percutaneous transluminalangioplasty, vascular access

## Abstract

Hemodialysis (HD) remains the cornerstone therapy for end-stage renal disease (ESRD), with arteriovenous fistulas (AVFs) generally favored over arteriovenous grafts (AVGs). Nonetheless, AVGs serve as a critical alternative for patients in whom AVF creation is not feasible. AVGs, however, carry risks such as infection, thrombosis, aneurysm formation, steal syndrome, and graft-to-vein fistulas (GVFs). We report the case of a 62-year-old patient with ESRD who developed partial thrombosis secondary to an inadvertent GVF during AVG cannulation. Remarkably, the GVF was preserved as a secondary HD access and remained functional for more than a year without major complications. This case highlights the potential role of GVFs as a viable option in HD access management.

## Introduction

The global prevalence of end-stage renal disease (ESRD) continues to rise. Data from the China National Hemodialysis and Peritoneal Dialysis Registry indicate that 243,863 patients underwent HD in 2012(1), whereas 184,122 individuals initiated HD in the United States during 2023–2024 (2). Over the past six decades, vascular access for HD has predominantly comprised AVFs, AVGs, and central venous catheters (CVCs). Among these, AVFs are generally preferred because they are associated with lower rates of infection and thrombosis as well as superior long-term patency(3). In contrast, AVGs are indispensable for patients in whom AVF creation is not feasible. Sustaining reliable vascular access is critical for patient survival, yet access-related complications remain a major source of morbidity and mortality. AVGs are particularly prone to complications, including infection, aneurysm formation, thrombosis, steal syndrome, and iatrogenic fistula development (4, 5). Iatrogenic fistulas can impair the function of AVGs by diverting blood flow and increasing the risk of recurrent thrombosis. We present the case of an AVG complicated by a functional iatrogenic GVF that developed during cannulation. Rather than being ligated, the GVF was preserved and successfully utilized as a secondary HD access. This case highlights important considerations regarding GVF pathogenesis, management, and strategies to reduce the risk of partial thrombosis.

## Case description

A 62-year-old patient with ESRD had been receiving hemodialysis for two years. Due to the absence of suitable superficial veins, the patient was not eligible for AVF creation and underwent surgical placement of a left brachiocephalic AVG using expanded polytetrafluoroethylene (ePTFE). The graft thrombosed shortly after implantation, prompting revision surgery in which the arterial limb was anastomosed to the ulnar artery.

**Figure 1:**
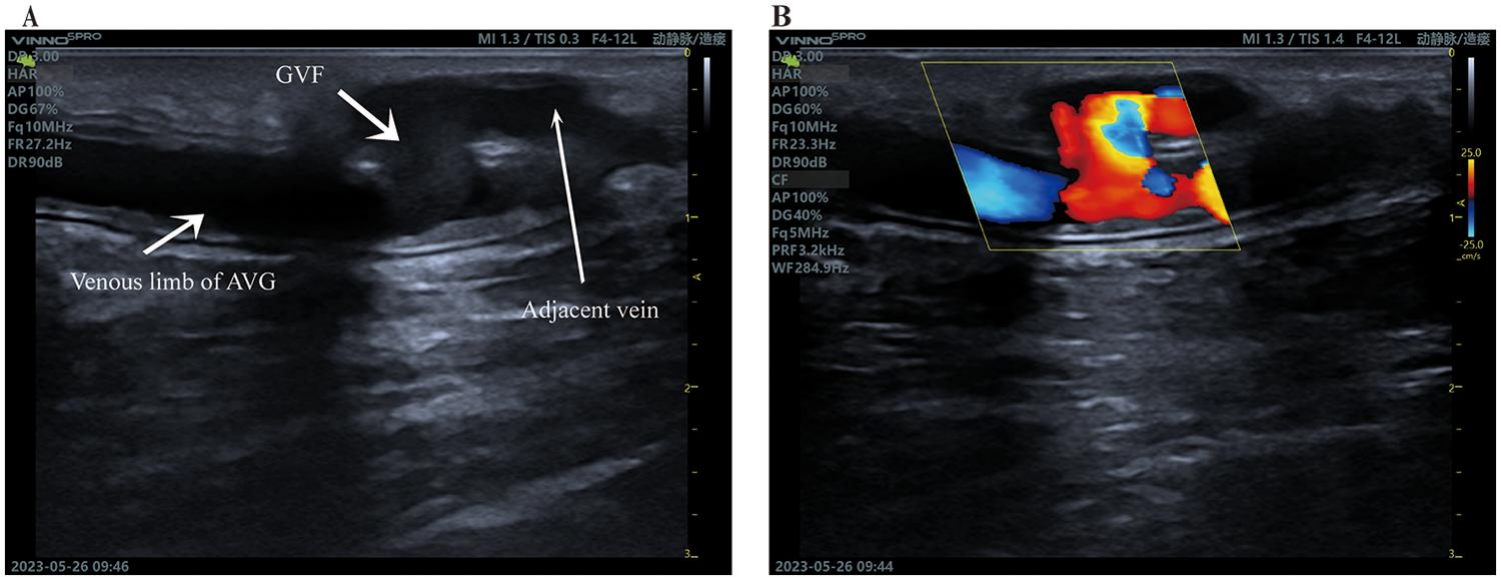
Graft-vein fistula. **(A)**, Graft-vein fistula between the venous limb and an adjacent vein. **(B)**, blood flow through the GVF.

**Figure 2:**
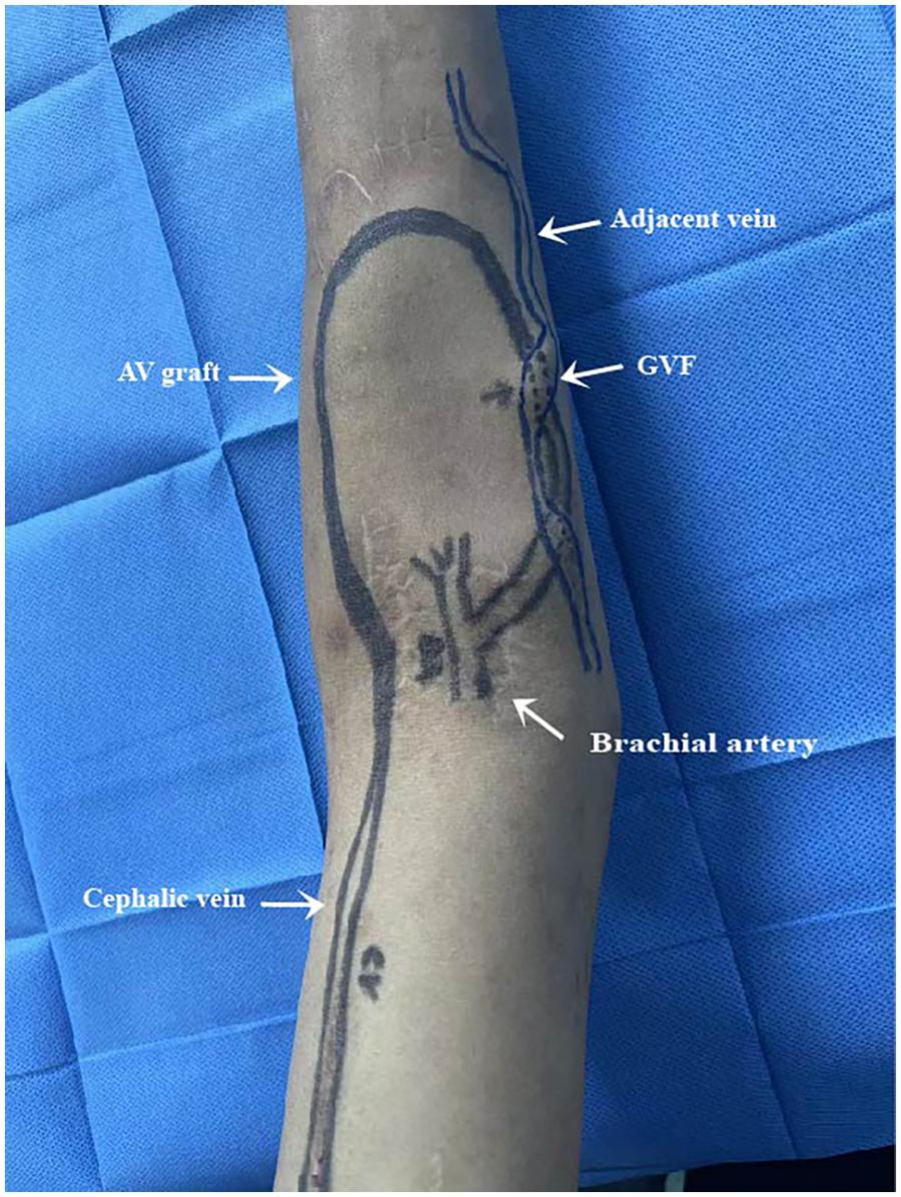
Diagrammatic representation of Duplex identification of brachial artery, brachial vein, and graft-vein fistula (GVF) with relevant dimensions.

Eight months later, the patient presented with diminished thrill at the access site, suggesting dysfunction. Doppler ultrasound revealed an embolic event in the venous segment of the AVG, and an iatrogenic GVF connecting the arterial portion of the graft to adjacent veins (Figures 1, 2). Percutaneous transluminal angioplasty (PTA) with a 6-mm balloon was performed on the venous segment and cephalic vein. Fistula ligation was deferred, given its degree of blood flow shunting was insufficient to significantly compromise AVG function after PTA.

**Figure 3:**
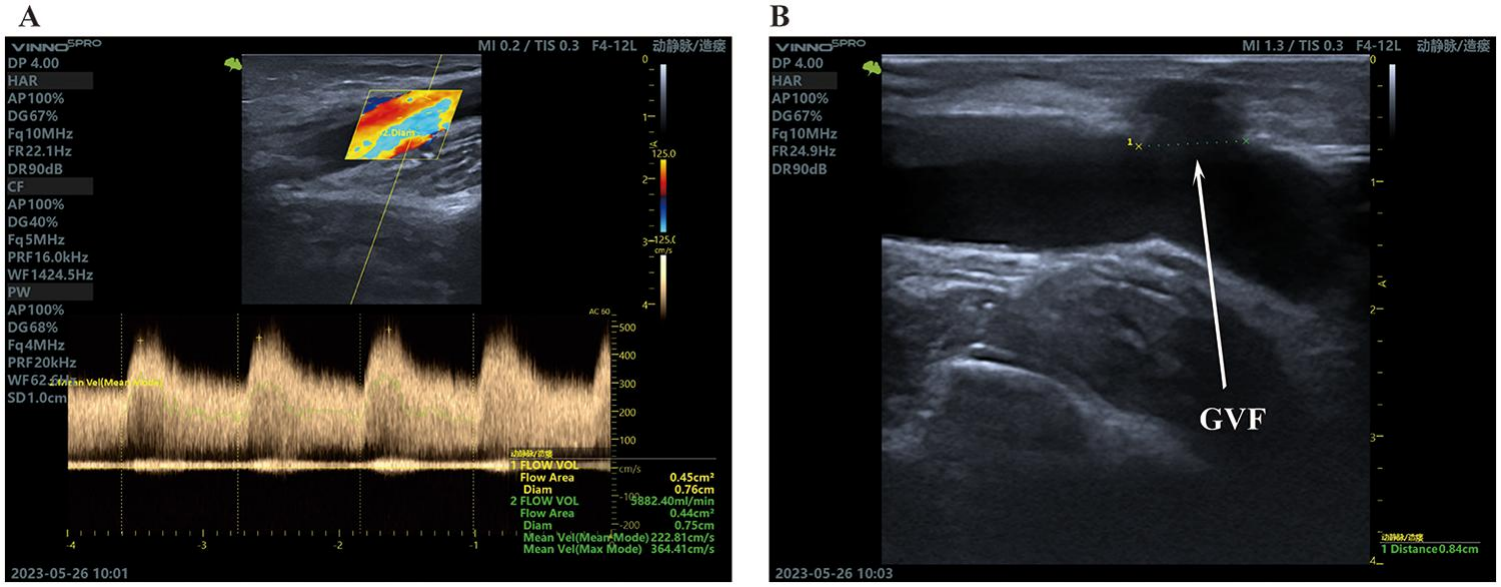
GVF as an alternative outflow. **(A)**, The flow area is 0.45 cm^3^, flow vol is 5,882.40 mL/min. The brachial artery flow is 1,282 mL/min, the resistance index (RI) is 0.33, and the blood flow during HD is 240 to 260 mL/min. **(B)**, GVF diameter is 8.4 mm.

At one-month follow-up, thrombosis recurred in the venous segment, while the GVF maintained a palpable thrill, indicating continued patency (Figure 3). Ultrasound confirmed a patent GVF measuring 8.4 mm in diameter without outflow stenosis. The brachial artery demonstrated a flow rate of 1,282 mL/min with a resistance index (RI) of 0.33. During hemodialysis, both the arterial segment of the graft and the GVF were successfully utilized as access points, supporting blood flow rates of 240–260 mL/min. No antiplatelet or anticoagulant therapy was administered. At the time of reporting, the GVF had remained functional for over one year and continues to serve as an effective hemodialysis access.

## Discussion

Hemodialysis grafts are associated with several potential complications, including thrombosis, infection, steal syndrome, and the formation of pseudoaneurysms, which carry a risk of hemorrhage. Other recognized issues include venous hypertension, the development of seromas, and, in some cases, cardiac overload leading to heart failure (4). Thrombosis and infection are more commonly observed in AVGs than in AVFs. A GVF refers to an abnormal vascular connection between an AVG and neighboring veins. This phenomenon was initially described by Dousset et al., who identified GVFs in 2 out of 22 patients with hemodialysis grafts using color Doppler imaging. (6). Kanterman et al. reported five cases of abnormal fistulous connections between dialysis grafts and adjacent native veins. All instances were associated with venous anastomotic stenosis or outflow obstruction. Venous stenosis is recognized as a key pathogenic factor in graft thrombosis, as the narrowed fistulous lumen fails to adequately reduce distal circuit blood flow, thereby promoting thrombus formation. During follow-up, all five grafts remained functional, and no additional complications were observed (7). Several case reports (8–10) have described instances in which partially thrombosed polytetrafluoroethylene (PTFE) grafts were preserved due to spontaneous fistulization into nearby forearm veins. After reviewing these cases, investigators emphasized four key aspects of this abnormal fistula: its distinctive presentation, incidence, etiology, and management. Clinical findings suggestive of a graft-to-vein fistula (GVF) with partial thrombosis include a palpable thrill confined to the arterial limb, dilated superficial forearm veins, thrombosis restricted to the venous limb, and aspiration of pulsatile arterial blood from the arterial side with dark blood or clot from the venous side. GVF should be suspected when routine graft surveillance reveals abnormalities such as elevated venous pressure, increased recirculation, or reduced dialysis adequacy (KT/V). Duplex ultrasonography may demonstrate blood flow limited to a segment of the graft and abnormal connections to adjacent veins, whereas angiography remains the gold standard for definitive diagnosis, as it visualizes contrast medium traversing from the graft into neighboring veins through the fistulous connection. In the present case, clinical evaluation revealed a palpable thrill restricted to the arterial limb and thrombosis confined to the venous segment of the graft—findings strongly indicative of a GVF. This preliminary suspicion was subsequently confirmed by ultrasonographic assessment.

Standage's report also addressed the incidence of graft-to-vein fistulas (GVFs), citing rates of approximately 9%, 3.9%, and as low as 0.027% in previously published case studies. In China, where the dialysis population exceeds 700,000, fewer than 3% rely on arteriovenous grafts (AVGs) for hemodialysis access (11). Clinical experience with GVFs in China remains limited, making it difficult to establish their true incidence. Although the actual prevalence may be underestimated, likely due to the small caliber of these fistulous connections. Previous reports have emphasized venous outflow stenosis as a major contributor to graft-to-vein fistula (GVF) formation, suggesting it should be the primary target for correction. In contrast, Seung et al. (12) described two cases of GVF with thrombosis in South Korea that were managed with fistula ligation, arguing that GVF itself precipitates AVG thrombosis by reducing graft blood flow. Whether venous outflow stenosis or diminished graft flow plays the dominant role in AVG thrombosis remains controversial across different studies. Notably, in our case, repeat assessment revealed no evidence of venous outflow stenosis despite recurrent thrombosis in the venous limb of the AVG. These findings support the interpretation that shunting of blood from the AVG into the GVF was the primary mechanism underlying graft thrombosis in this patient. Haddad et al. (12) reported five cases of GVF formation, noting that in case 3 the GVF resolved spontaneously following PTA, while in case 4 it was no longer evident at the time of thrombectomy. These observations suggest that GVF may, in some instances, represent a natural “cure.” However, other reports describe GVF occurring in the context of established thrombosis or graft infection. Importantly, GVF has also been proposed as an alternative therapeutic strategy to thrombectomy or complete graft excision(13, 14).

The exact mechanism underlying GVF formation remains uncertain; however, repeated traumatic cannulation is thought to play a central role. When intragraft pressure is not elevated by pre-existing venous outflow stenosis, GVFs may resolve spontaneously over time. Continuous monitoring of blood flow in both the GVF and the AVG is therefore essential to guide management and preserve vascular access. In our case, the presence of the GVF was identified only after thrombosis developed in the arteriovenous graft AVG. The surgical team considered that thrombosis in the AVG was primarily attributable to narrowing of the venous outflow tract. Blood flow through the GVF was insufficient to significantly alter overall graft hemodynamics. Based on prior case reports, the treatment team elected to first address venous outflow tract stenosis with PTA, followed by close ultrasound surveillance of both the AVG and GVF to determine whether ligation would be necessary. This experience led us to conclude that when an AVG is complicated by GVF and upper-limb swelling cannot be explained by other causes—such as pseudoaneurysm formation or graft structural compromise—intervention should be considered. Specifically, PTA and surgical ligation of the GVF simultaneously may be warranted to prevent recurrent graft thrombosis.

Following the initial PTA, stenosis of the AVG venous outflow tract recurred. As the stenosis progressed, partial thrombosis repeatedly developed within the AVG over the course of one month. The resulting obstruction increased intragraft pressure, which in turn enlarged the GVF diameter and promoted its persistence. Owing to the patient's unique venous anatomy, the GVF vessels were located in a relatively superficial subcutaneous position, making them suitable for dialysis access. The vessel diameter was sufficiently large, with no evidence of stenosis or thrombosis. Color Doppler ultrasound confirmed that the GVF functioned effectively as an alternative hemodialysis outflow pathway, demonstrating favorable blood flow characteristics. In addition, the arterial segment of the AVG and adjacent veins provided sufficient length and accessibility, ensuring reliable dialysis cannulation. Quan Zheng et al. reported postintervention patency rates for AVGs of 78.5%, 57.4%, and 20.0% at 3, 6, and 12 months, respectively—substantially lower than those observed for AVFs(15). In our case, AVG patency lasted less than one month following PTA, suggesting that repeat intervention would likely yield similarly limited results. Had the graft failed again, the patient would have required a central venous catheter (CVC), which carries increased risks of infection and central venous thrombosis. Given that Doppler assessment confirmed adequate blood flow and the patient experienced no discomfort during dialysis, the GVF was maintained as the primary access without further intervention. Remarkably, the GVF has remained patent for over one year without complications such as stenosis or thrombosis.

This experience suggests that while GVFs may contribute to graft thrombosis under certain conditions, they can also serve as a viable alternative access route for sustained hemodialysis when closely monitored. Prevention of GVF formation relies heavily on proper cannulation technique, including avoiding puncture sites near adjacent veins, rotating cannulation sites to allow tissue healing, and ensuring adequate compression after each session to prevent persistent abnormal connections. If a GVF is detected without associated thrombosis, early ligation is advisable. When GVF coexists with thrombosis, ultrasound assessment of fistula diameter and blood flow is critical. Adequate flow and the absence of venous stenosis may justify preserving the GVF as an alternative access, whereas otherwise, ligation following PTA should be pursued.

In conclusion, this report describes a case of partial thrombosis in an arteriovenous graft complicated by a graft-to-vein fistula, managed successfully through routine follow-up without additional PTA or fistula ligation. This case adds to the limited literature on GVF, offering further insight into the management of this rare complication and its potential role as an alternative access pathway.

## Data Availability

The original contributions presented in the study are included in the article/Supplementary Material, further inquiries can be directed to the corresponding authors.
